# Cognitive load and individual differences as drivers of health-related misinformation: A signal detection approach

**DOI:** 10.1371/journal.pone.0356642

**Published:** 2026-08-28

**Authors:** Guillermo Puebla, Rodrigo Ferrer-Urbina, Daniel Pérez-Zapata, Karina Alarcón-Castillo, Geraldy Sepúlveda-Páez, Herman Elgueta

**Affiliations:** 1 Escuela de Administración y Negocios, Universidad de Tarapacá, Arica, Chile; 2 Escuela de Psicología y Filosofía, Universidad de Tarapacá, Arica, Chile; 3 School of Psychology, University of Birmingham, Birmingham, United Kingdom; 4 Departamento de Psicobiología y Metodología en Ciencias del Comportamiento, Universidad Complutense de Madrid, Madrid, Spain; 5 Departamento de Psicología, Universidad de Magallanes, Punta Arenas, Chile; University of Haifa, ISRAEL

## Abstract

The global spread of health-related misinformation during the COVID-19 pandemic has highlighted the need to understand the psychological mechanisms that make individuals susceptible to health-related misinformation. This study examined the impact of cognitive load and individual differences on susceptibility to COVID-19 misinformation using a Signal Detection Theory framework. A sample of 634 university students evaluated true and false COVID-19 headlines under working memory load and no-load conditions, while also completing standardized assessments of cognitive reflection, epistemically unwarranted beliefs, naïve skepticism, and bullshit receptivity. Results showed that, although the working memory load manipulation successfully impaired recall and increased response time, it did not affect participants’ tendency to accept false information as true. In contrast, individual-difference variables robustly predicted misinformation susceptibility. Specifically, beliefs in pseudoscience, epistemically unwarranted beliefs, naïve skepticism, and bullshit receptivity showed moderate positive associations with false-alarm rates and negative associations with discrimination sensitivity. Cognitive reflection was negatively associated with false-alarm rates and positively with discrimination sensitivity. Only small associations were found between individual-difference dimensions and response bias. These findings suggest that stable cognitive and epistemic dispositions play a significant role in shaping people’s ability to distinguish between accurate and false health information.

## Introduction

At the beginning of the pandemic, why did individuals use chlorine dioxide products for COVID-19 protection, even though there was no evidence confirming the efficacy of industrial disinfectants as a cure for the virus? Why are groups of people reluctant to be inoculated against this virus all over the world? Health-related misinformation can rapidly spread globally, influencing people’s behavior and potentially hampering their efforts to protect themselves, for example, from the coronavirus [1,2]. In this scenario, a fundamental question arises: What psychological factors contribute to an individual’s susceptibility to health-related misinformation? In this study, our aim was to explore how cognitive load and several individual-difference dimensions affect individuals’ tendency to fall for COVID-19 misinformation.

Broadly defined, misinformation is any information given to the public that turns out to be false [3]. The spread of misinformation is a serious challenge to public health and has hindered global efforts to manage the COVID-19 pandemic [1]. Indeed, the overload of frequently false and misleading information, known as an “infodemic”, has had a large negative impact on people’s health-protective behavior [4]. For example, the dissemination of misinformation about COVID-19 among the general public has revealed a marked lack of adherence to public health guidelines [5–7]. Furthermore, projections from social network analysis suggest that, if no action is taken, anti-vaccination content on social media will predominate in discussions over the next decade [8]. For these reasons, understanding the psychological drivers of misinformation susceptibility is crucial to tackling several challenges across various aspects of our society (e.g., disbelief in democracy, climate change action, and vaccination), especially for maintaining health-protective behaviors. To understand these psychological drivers, however, it is necessary first to define misinformation susceptibility.

### Characterizing misinformation susceptibility: Signal detection theory

In this work, we used Signal Detection Theory (SDT) [9] to characterize the different facets of misinformation susceptibility. SDT is a mathematical framework originally developed in the context of sensory psychology to disentangle how different factors influence people’s ability to discriminate between true information (signal) and misinformation (noise). Applied to the study of misinformation susceptibility, SDT distinguishes two distinct aspects in the identification of fake news: (1) the ability to distinguish between real news and fake news, and (2) response biases to judge news as real or fake regardless of veracity [10]. From this point of view, understanding why people are susceptible to believing false information involves analyzing how they categorize true and false statements. SDT identifies four possibilities: correctly identifying true information (hit), correctly identifying false information (correct rejection), incorrectly identifying true information as false (miss), and incorrectly identifying false information as true (false alarm). From this perspective, the problem of misinformation susceptibility is equivalent to identifying the causes of false alarms [11–13].

As stated, SDT suggests two main reasons for false alarms. First, people might struggle to distinguish accurate from inaccurate information, leading to more false alarms and more misses. This difficulty would manifest as a low discrimination sensitivity index, $d^{\prime }$, which measures the ability to discriminate between true and false information. Discrimination sensitivity is defined as:


$$\begin{array}{c} d^{\prime }=z(\text{H})-z(\text{FA}) \end{array}$$ (1)

Where H is the proportion of hits and FA is the proportion of false alarms. In this equation, both H and FA are transformed into *z*-scores using the inverse cumulative distribution function such that a proportion of 0.5 is converted to a z-score of 0 [14]. Thus, a $d^{\prime }$ of zero indicates chance-level performance, and higher scores reflect greater accuracy.

Second, people may tend to believe information is true, regardless of its veracity. This would result in a high number of both hits and false alarms, reflected in a low response bias index *c*, which indicates the threshold for judging information as true or false. Response bias is defined as:


$$\begin{array}{c} c=-\frac{z(H)+z(FA)}{2} \end{array}$$ (2)

A *c* of zero signifies no bias, a positive *c* indicates a bias towards judging information as false, and a negative *c* indicates a bias towards judging information as true. Although misinformation susceptibility might be driven by a general tendency to accept all information as true, it seems more likely that this depends on the congruence of the new information with respect to prior beliefs [12]. According to SDT, this would likely be reflected in a lower *c* index (indicating a stronger bias toward belief) for belief-congruent information relative to belief-incongruent information [10].

Taking an SDT approach allows us to investigate how several psychological factors affect people’s tendency to accept false information as true, as well as characterize this influence. In particular, we seek to identify whether each psychological factor in our study that influences people’s false alarm rates also

### Psychological drivers of misinformation

What makes some people more susceptible to belief in health-related misinformation? One influential view suggests that misinformation susceptibility is the result of a lack of engagement in analytic reasoning [15]. For example, according to dual-process theories, human reasoning is composed of two distinct types of processes: Type 1 and Type 2 [16–18]. On the one hand, Type 1 processing is characterized as automatic, with intuitive answers quickly coming to mind in response to a given stimulus. Importantly, judgments based on Type 1 processing are accurate most of the time, but can lead to mistakes in certain cases. In contrast, Type 2 processing is characterized as deliberate, which tends to result in a more rigorous evaluation of information —but this system can also lead to inaccurate responding [16]. Research in the dual-process tradition has shown that, in general, misinformation acceptance is most likely to occur when intuitive and autonomous processes are elicited [19]. For example, when people are exposed to a large volume of information in a short period, Type 1 processing is more likely to be activated, which could make them more prone to trusting misinformation [20]. Conversely, when individuals have the opportunity to think analytically and carefully about a piece of information (i.e., when Type 2 processing is active), they tend to be more successful at identifying false information [21]. Accordingly, in this work, we investigated the effect of cognitive load on susceptibility to health-related misinformation. We hypothesize that loading participants’ working memory will increase the false alarm rate.

In addition to external influences on analytic reasoning, the individual-difference dimension of cognitive reflection also plays a critical role in susceptibility to misinformation. This refers to the capacity to resist intuitive responding and instead engage in deliberate thought when evaluating information [22]. A substantial body of research has demonstrated that individuals who score higher in cognitive reflection are significantly more accurate at distinguishing real news from fake news [15,23–26]. Notably, some studies suggest that encouraging conscious deliberation can reduce susceptibility to fake news even when controlling for individuals’ partisan affiliations [21]. Based on these findings, we hypothesize that cognitive reflection will be negatively associated with false-alarm rates and positively associated with discrimination sensitivity.

Another influential account, known as motivated reasoning, is based on the idea that people’s susceptibility to misinformation is not due to a faulty process of careful consideration, but rather to starting their reasoning with a pre-set goal, which leads them to interpret new information in service of reaching that goal [1,27,28]. For example, some people wrongly believe that the deployment of 5G technology is linked to the spread of the coronavirus, and they frame new information in line with this belief [29]. Some beliefs could be considered especially effective “sources” for motivated reasoning. In particular, pseudoscience, conspiracy theories, and paranormal beliefs are collectively referred to as epistemically unwarranted beliefs [30,31]. The interconnectedness of these beliefs highlights the shared attributes among pseudo-scientific, conspiratorial, and paranormal thoughts [30]. For example, individuals with a predominant disposition to believe in epistemically unwarranted beliefs may be highly motivated to accept misinformation, such as claims that the SARS-CoV-2 virus was a bioweapon artificially created by the Chinese government [32] or intentionally created by powerful people [33]. Based on these considerations, we hypothesized that pseudoscience, conspiracy theories, and paranormal beliefs are positively correlated with false alarm rates and negatively associated with discrimination sensitivity.

Beyond investigating psychological factors related to the analytic reasoning and motivated reasoning accounts, we also included two additional individual-difference measures. The first one is naïve skepticism. This mindset is characterized by individuals’ tendency to disregard information without applying critical thinking or evidence-based reasoning, rejecting claims simply to be skeptical [34,35]. Naïve skeptics not only tend to question official sources of information (e.g., mainstream media outlets, scientific and governmental organizations) but are also particularly vulnerable to believing misinformation [35,36]. It has been pointed out that naïve skeptics tend to disregard information that is inconsistent with their idiosyncratic and group identities [37]. From an SDT point of view, this tendency entails a low veracity threshold for belief-congruent information, which should lead to more false alarms. At the same time, this tendency entails a high veracity threshold for belief-incongruent information which should lead to more misses. Notably, these two effects can only happen simultaneously if the discrimination sensitivity is low. Based on these considerations, we hypothesized that naïve skepticism is negatively associated with discrimination sensitivity.

The second one is bullshit receptivity, which refers to the tendency to perceive statements as deep or significant, even when they are in fact nonsensical or meaningless [38]. Concretely, people with higher levels of bullshit sensitivity might interpret abstract language as coherent, even when it lacks any depth or clarity. For example, consider the following abstract phrase: “The invisible dance of the universe whispers truths beyond reality”. People with higher levels of bullshit sensitivity may perceive the statement as conveying a deeper metaphysical insight, even though the phrase itself is ambiguous and lacks clear meaning. Previous research on political misinformation has found negative associations of bullshit receptivity with analytical thinking [25] and response bias [39]. Based on this evidence, we hypothesize that bullshit sensitivity should be positively associated with false alarm rate and negatively with response bias.

### The current research

The present study had two main goals. In the first place, we sought to gauge the influence of working memory load on people’s susceptibility to misinformation. While previous research has used time pressure to manipulate cognitive elaboration [11,12,21,40], we induced working-memory load more directly through a secondary digit recall task. Similarly to previous research examining the effects of working memory load on reasoning processing and decision making [41,42], the current work compared the credibility that people assign to health-related misinformation in two scenarios: one taxing working memory (i.e., inducing Type 1 processing) and the other involving open or free processing (i.e., facilitating Type 2 processing). More concretely, participants rated 32 COVID-19 headlines as true or false. In the working memory load phase, participants received a 5-digit number to remember before rating each COVID-19 statement, and were then asked to recall it as accurately as possible after evaluating the statement. In the no working memory load phase, participants received and recalled the 5-digit number before evaluating each COVID-19 statement.

In the second place, the current study investigated whether the individual-difference dimensions cognitive reflection, epistemically unwarranted beliefs, naïve skepticism, and bullshit receptivity were associated with misinformation susceptibility. Specifically, we analyzed the relationships between these individual-difference dimensions and false-alarm rate, discrimination sensitivity, and response bias. To summarize, our hypotheses are as follows:

1. Loading working memory increases false alarm rates on the fake news identification task.
2. Cognitive reflection is negatively associated with false alarm rates and positively associated with discrimination sensitivity.
3. Epistemically unwarranted beliefs are positively associated with false alarm rates and negatively associated with discrimination sensitivity.
4. Naïve skepticism is negatively associated with discrimination sensitivity.
5. Bullshit receptivity is positively associated with false alarm rates and negatively with response bias.

## Methods

### Ethics

Ethical approval was obtained by the Ethics Committee of the University of Tarapacá (reference number N°14/2022, internal codes CEC C11-2022 and CEC C12-2023) prior to the start of the study. Written informed consent was obtained from all individual participants prior to their participation in the study. All procedures performed with human participants were in accordance with the ethical standards as laid down in the 1964 Declaration of Helsinki and its later amendments or comparable ethical standards.

### Participants

We calculated the initial sample size using the sofware Gpower 3.1 [43]. Assuming a small effect size of 0.15, 2 repeated measures, 2 covariates (naïve skepticism and bullshit receptivity), a type I error of α=0.01, and a statistical power of 1−β=0.95, we estimated a sample of a sample of 598 participants (299 for each of the 2 starting conditions). Additionally, we planed to recruit 10% more participants to compensate for potential sample losses. Our actual sample consisted of a total of 634 people working or studying at one of 5 universities in Chile (Universidad de Tarapacá, Universidad de Talca, Universidad de Católica del Norte, Universidad de Chile, and Universidad de Magallanes) participated in return for approximately 8 dollars. The mean age was 21.9 years (SD 4.42). Gender wise, 57.2% (*n* = 363) identified as female, 39.4% (*n* = 250) as male, and 3.4% (*n* = 22) as non-binary or other. Education-wise, 93.9% (*n* = 596) were university students. Regarding religion status 46.8% (*n* = 289) declared to be Catholic or Christian, and 53,2% (*n* = 329) identified as agnostic, atheist or without religion. Regarding political tendency, 49.6% (*n* = 289) identified as left lining, 8.2% (*n* = 48) as center lining, 15.6% (*n* = 91) as right lining, and 26.6% (*n* = 155) as apolitical. Data collection took place between 28 August 2023 and 15 December 2023 and this study received ethical approval from the University of Tarapacá Ethics Committee.

### Materials

#### COVID-19 headlines.

The 32 headlines used in the experiment were taken from Elgueta et al. [44], who drew a corpus of 240 COVID-19 true and false news items compiled and verified by the Chilean fact-checking platforms Fast Check (https://www.fastcheck.cl/) and Mala Espina (https://www.malaespinacheck.cl/). From this corpus, we selected 32 headlines to represent the main thematic categories identified in that study. These themes included items related to the nature of the pandemic (e.g., supposed cures, virus characteristics), preventive measures (particularly vaccines and masks), and alternative narratives (e.g., conspiracy-oriented claims). Half of the headlines were true statements (e.g., “Masks are effective in preventing the spread of COVID-19”) and half were false (i.e., “COVID-19 vaccine may cause infertility”). The full list of headlines used in this study is provided in S1 File.

#### 5-digit recall task.

Our working memory loading task consisted of recalling randomly generated 5-digit numbers (e.g., “45638”) after a short delay. In the working memory load condition, participants were presented with a 5-digit number before evaluating each COVID-19 headline and were required to recall it immediately afterward. In the no working memory load condition, participants were presented with a 5-digit number and had to recall it before evaluating each COVID-19 headline. All participants completed both conditions (i.e., a within-subjects design).

#### Cognitive reflection test.

Version developed and extended from the original test proposed by [22] and the CRT-2 developed by [45]. This test assesses people’s ability to suppress automatic responses that seem obvious to find the correct answer. This version corresponds to a maximum-performance test comprising 7 items, extracted from both versions and adapted to the local and cultural context of our target population. For the adaptation, factors such as the use of local currency, the replacement of unfamiliar cultural references with more recognizable ones, and the adaptation of names and situations to make them more natural and closer to the target population were considered. Scores were calculated by computing the mean across all items. Measurement-model results, including factor loadings, are provided in S2 File.

#### Epistemically unwarranted beliefs scale.

Developed by [46], this instrument assesses the adherence to epistemically unwarranted beliefs in the general population. This scale has three dimensions: pseudoscience, conspiracy theories and paranormal beliefs, with 4 items each. Responses were measured using 5-point rating scales ranging from 1 (Strongly disagree) to 5 (Strongly agree). Scores were calculated by computing the mean across all items.

#### Naïve skepticism scale.

Developed by [47], this instrument assesses the level of naïve skepticism in the general population. This scale has two dimensions: skepticism towards science (henceforth, naïve skepticism science; 7 items) and skepticism towards governmental organizations and the official press (henceforth, naïve skepticism government; 7 items). Responses were measured with 5-point rating scales ranging from 1 (Never) to 5 (Always). Scores were calculated by computing the mean across all items.

#### Bullshit receptivity scale.

Developed by [48], this instrument assess people’s tendency to assign meanings to randomly generated pseudo-profound sentences and to differentiate them from sentences with intentional meaning. This instrument has two dimensions: susceptibility to nonsense (henceforth, bullshit receptivity; 8 items) and sensitivity to sense (6 items). Given that we were interested in people’s tendency to find meaning in pseudo-profound sentences, we only used the first dimension of the instrument. Responses were measured with 4-point rating scales ranging from 1 (Nonsense) to 4 (A lot of sense). Scores were calculated by computing the mean across all items.

### Procedure

Participants started by completing the COVID-19 headlines evaluation task and the 5-Digit recall task. As explained earlier, there were two within-participant conditions: working memory load and no working memory load. The COVID-19 headlines were randomly assigned to both conditions (i.e., 16 per condition, half true, half false) for each participant. The order of these conditions was counterbalanced across participants. After completing the main experimental task, participants answered the questionnaires. Our study was implemented in Psychopy^®^ [49] and is available in: https://anonymous.4open.science/r/health_missinfo_exp-7CDC.

### SDT indexes calculation

For each participant, we calculated hit rates (H) as the proportion of true headlines judged as true and false-alarm rates (FA) as the proportion of false headlines judged as true. When a participant gave no true responses (a proportion of 0), we followed the recommendations of [50] and converted the values to 1/(2×N), where *N* is the number of headlines. Similarly, if the proportion of true responses was 1, we converted values to 1−1/(2×N). Discrimination sensitivity scores were calculated using Eq (1) and response bias was calculated according to Eq (2).

### Data analysis

To characterize the study variables, we conducted descriptive analysis of central tendency and dispersion. We then analyzed participants’ performance using a generalized linear mixed-effects model. Subsequently, conducted a Welch’s t-test to evaluate the effect of the working memory load task on participants’ digit recall performance and reaction times to the COVID-19 headlines. The measurement models of the individual differences instruments were tested using exploratory structural equation modeling (ESEM) with GEOMIN rotation [51] and, for CRT, which is unidimensional, confirmatory factor analysis (CFA). These models were estimated from the polychoric correlation matrix using the weighted least squares WLSMV estimation method, which is robust to non-normal discrete variables and performs adequately for ordinal variables [52]. After establishing the measurement models’ goodness of fit, we calculated latent-variable correlations between our individual-difference dimensions and the false-alarm rate, discrimination sensitivity, and response bias, while restricting covariances among latent variables to zero. We used Cohen’s [53] recommended *r* values of 0.10, 0.30, and 0.50 to demarcate small, medium, and large effects, respectively. Finally, to evaluate the joint influence of our individual-difference dimensions on the false-alarm rate, discrimination sensitivity, and response bias, we used structural equation modeling. These models were estimated with the weighted least squares WLSMV estimation method. The fit of these models was assessed following the cut-point recommendations proposed by [54] for the comparative fit index (CFI), Tucker–Lewis index (TLI), and root mean squared error of approximation (RMSEA): CFI and TLI > 0.90 is acceptable and >0.95 is satisfactory; RMSEA < 0.08 is acceptable and <0.06 is satisfactory.

## Results

Table 1 presents the means and standard deviations of the proportions of hits, false alarms, misses, correct rejections, and SDT measures discrimination sensitivity ($d^{\prime }$) and response bias (*c*) using the items of both the working memory load and no working memory load conditions. As can be seen, in our sample the proportion of false alarms was low but significantly different from zero *t*(634) = 23.2, *p* < .001, *d* = 0.919, and the proportion of hits was high and significantly different from zero *t*(634) = 228.2, *p* < .001, *d* = 9.057. Regarding the SDT measures, discrimination sensitivity was positive and significantly different from zero, *t*(634) = 82.18, *p* < .001, *d* = 3.261, and response bias was also significantly different from zero, *t*(634) = 7.85, *p* < .001, although as can be seen in Table 1 it was close to 0 (and the effect size was small *d* = 0.312).

**Table 1:** Rates of hits, false alarms, misses, correct rejections and SDT measures.

Variable	M	SD
Hits	0.908	0.100
False Alarms	0.092	0.100
Misses	0.118	0.093
Correct Rejections	0.882	0.094
Discrimination Sensitivity ($d^{\prime }$)	2.830	0.867
Response Bias (*c*)	0.086	0.275

### 5-digit recall task

To assess whether our working memory load manipulation influenced participants’ performance, we compared the number of correctly recalled digits and the time to answer to the COVID-19 headlines across the working memory load and no working memory load conditions. First, a paired-samples t-test indicated that the average number of correctly recalled digits was lower in the working memory load (*M* = 4.21, *SD* = 0.658) condition than in the no working memory load (*M* = 4.91, *SD* = 0.155) condition (t(634)=−28.146, *p* < 0.001, *d* = 1.117). Second, another paired-samples t-test indicated that participants took longer to answer the COVID-19 headlines in the working memory load (*M* = 8.68, *SD* = 3.430) condition than in the no working memory load (*M* = 7.98, *SD* = 2.960) condition (*t*(634) = 5.466, *p* < 0.001, *d* = 0.217). These two results indicate that our manipulation of working memory load indeed affected participants’ performance.

### Linear mixed effects modeling

To analyze participants’ performance accounting for the nested structure of our working memory load experiment, in which each participant responded to 16 COVID-19 headlines within each load condition, we employed a generalized mixed-effects regression model with the logit link function. This model was fitted using the *glmer* function in the *lme4* package (Version 2.0.1) in R. In this model, the dependent variable was whether each response was correct or not. The model included working memory load (with vs. without load) as a fixed effect. We incorporated random intercepts and slopes for participant ID and item ID to capture variations across participants and problems. According to this analysis increasing working memory load did not significantly affect the likelihood of a correct response (β=−0.06, SE = 0.05, z=−1.14, *p* = .256).

### Effects of working memory load on false-alarm rates and SDT indices

**Fig 1:**
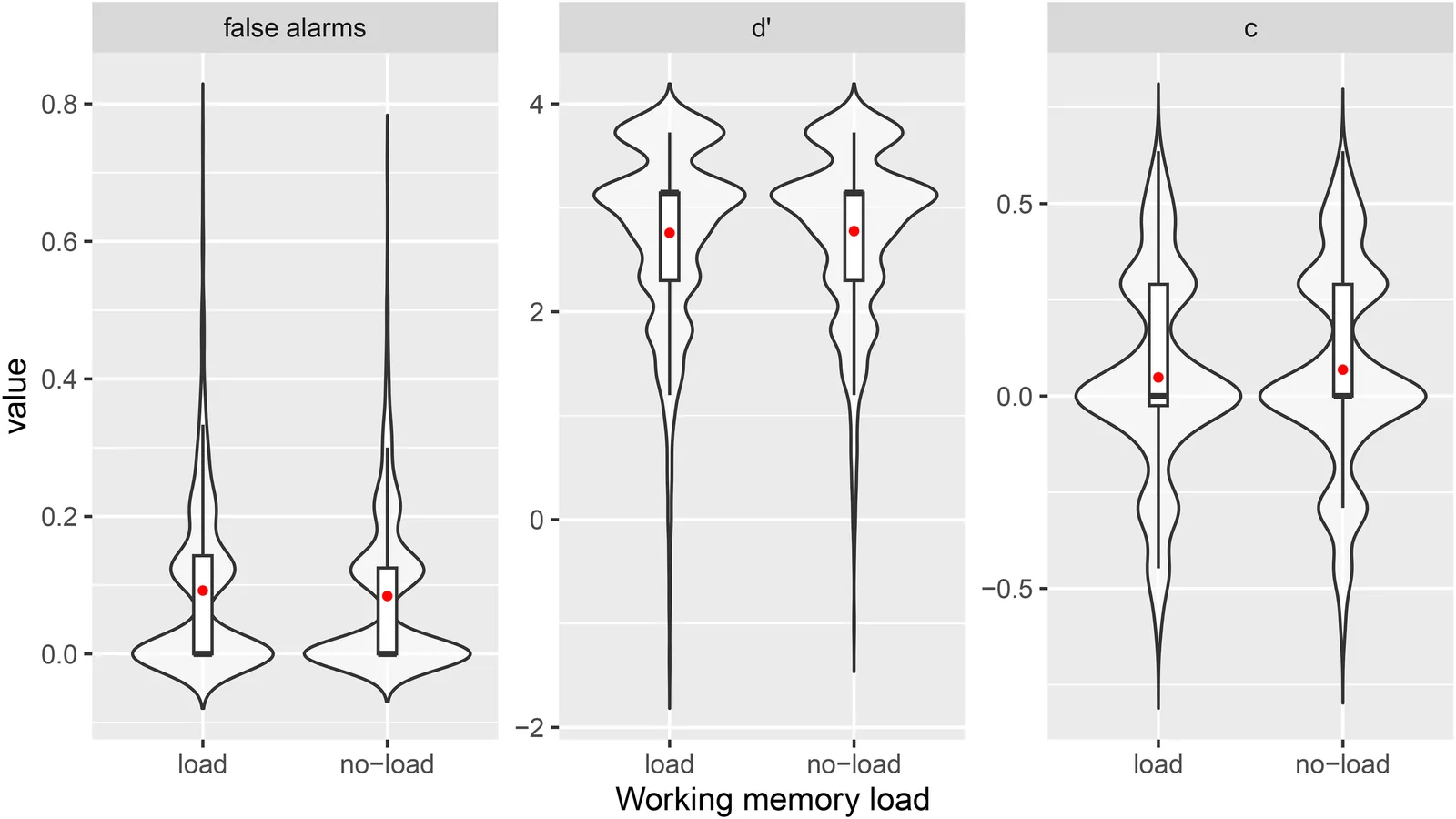
Effects of working memory load on false alarms rate, discrimination sensitivity, and response bias.

Regarding our main COVID-19 headlines judgment task, a paired-samples t-test showed that there was no difference in the proportion of false alarms between the working memory load (*M* = 0.092, *SD* = 0.121) and the no working memory load (*M* = 0.084, *SD* = 0.111) conditions (t(634)=−1.60, *p* = .110, d=−0.063) (Fig 1). A second paired-samples t-test showed that there was no difference in discrimination sensitivity between the working memory load (*M* = 2.76, *SD* = 0.858) and the no working memory load (*M* = 2.78, *SD* = 0.799) conditions (*t*(634) = 0.512, *p* = .609, *d* = 0.02. Finally, a third paired-samples t-test showed that there was no difference in response bias be*t*ween the working memory load (*M* = 0.05, *SD* = 0.262) and the no working memory load (*M* = 0.07, *SD* = 0.264) conditions (*t*(634) = 1.45, *p* = .147, *d* = 0.06. Overall, these results indicated that despite its effec*t*s on the number of correctly recalled digits and the time to answer the COVID-19 headlines, working memory load did not affect participants’ susceptibility to judge false COVID-19 information as true.

### Relations between false-alarm rates and SDT indices

Following previous research that used SDT to model misinformation susceptibility [11,13], we sought to replicate the effects of truth sensitivity and acceptance threshold on the acceptance of misinformation. Consequently, we conducted multiple-regression analyses using SDT indices $d^{\prime }$ and *c* to predict individuals’ false-alarm rates. As in previous research, to ensure the mathematical independence of predictors and the outcome, we calculated all scores separately for responses to odd-numbered and even-numbered headlines. We built two regression models, using responses to odd-numbered items as predictor variables and responses to even-numbered items as criterion variables, and vice versa. Our results indicated that acceptance of health-related misinformation showed significant negative associations with $d^{\prime }$ and *c* for both models (odd-to-even: *r*^2^ = .182, *F*(2,632)=70.5, *p* < .001, $\beta_{d^{\prime }}=-\mathrm{.401}$, $p_{d^{\prime }}<\mathrm{.001}$, $\beta_{c}=-\mathrm{.138}$, $p_{c}<\mathrm{.001}$; even-to-odd: *r*^2^ = .173, *F*(2,632)=65.9, *p* < .001, $\beta_{d^{\prime }}=-\mathrm{.375}$, $p_{d^{\prime }}<\mathrm{.001}$, $\beta_{c}=-\mathrm{.140}$, $p_{c}<\mathrm{.001}$). It is worth noting that the odd-even reliability of the SDT measures is *r* = .401 for $d^{\prime }$ and *r* = .207 for *c*. It is likely that the small variance of the proportion of hits and false alarms (see Table 1) and the number of COVID-19 headlines affected the reliability of the SDT measures.

### Relations between false-alarm rates and individual-difference dimensions

To establish the conditions for conducting latent variable analysis and structural equation modeling later, we first assessed the fit of the measurement models. As shown in Table 2, all measurement models exhibited adequate fit. Therefore, to identify the univariate effects of the 7 individual-difference measures on false-alarm rates, we computed the correlations among latent variables, restricting their covariances to zero. As can be seen in Table 3, this analysis showed medium direct effects in the case of pseudoscience and conspiracy theories, as well as small direct effects in the case of paranormal beliefs, naïve skepticism-science, naïve skepticism-government, and bullshit receptivity. Furthermore, we observed a small inverse effect on cognitive reflection.

**Table 2:** Measurement models fit.

Scale	$\chi^{2}$	*df*	$\chi^{2}/df$	RMSEA	90%CI	CFI	TLI	SRMR
CFA CTR	16.586*	14	1.184	.017	[.000, .044]	.996	.995	.050
ESEM EUB	74.746*	33	2.265	.045	[.031, .058]	.992	.984	.019
ESEM BR	213.825*	64	3.341	.061	[.052, .070]	.943	.919	.042
ESEM NS	200.489*	53	3.782	.066	[.057, .076]	.967	.951	.034

*Note*. *p < .001; CRT = Cognitive reflection test; BS = Epistemically unwarranted beliefs scale; BR = Bullshit receptivity scale; NS = Naive skepticism scale; RMSEA = Root mean square error of approximation; 90% CI = Confidence interval; CFI = Comparative fit index; TLI = Tucker-Lewis index; SRMR = Standardized root mean square residual.

**Table 3:** Latent variable correlations between individual difference dimensions and false-alarm rate, discrimination sensitivity, response bias.

Variable	False-Alarm Rate	Discrimination Sensitivity	Response Bias
Cognitive Reflection	−0.166*	0.184*	−0.023
Pseudoscience	0.398*	−0.366*	−0.175*
Conspiracy Theories	0.304*	−0.296*	−0.173*
Paranormal Beliefs	0.248*	−0.216*	−0.113
Naïve Skepticism Science	0.276*	−0.240*	−0.143*
Naïve Skepticism Government	0.282*	−0.249*	−0.122
Bullshit Receptivity	0.278*	−0.206*	−0.194*

*Note*. *p < .001.

### Relations between discrimination sensitivity and individual-differences dimensions

Similarly, we calculated a correlation of latent variables between the 7 individual-difference measures and truth sensitivity (see Table 3). As can be seen, we found a medium inverse effect in the case of pseudoscience and small inverse effects in the case of conspiracy theories, paranormal beliefs, naïve skepticism-science, naïve skepticism-government, and bullshit receptivity. Furthermore, we observed a small direct effect in the case of cognitive reflection.

### Relations between response bias and individual-Differences dimensions

We also calculated a correlation of latent variables between the 7 individual-difference measures acceptance threshold (see Table 3). This analysis showed only small inverse effects in the case of pseudoscience, conspiracy theories, naïve skepticism-science, and bullshit receptivity.

### Joint effects of individual differences dimensions on false-alarm rates and discrimination sensitivity

**Fig 2:**
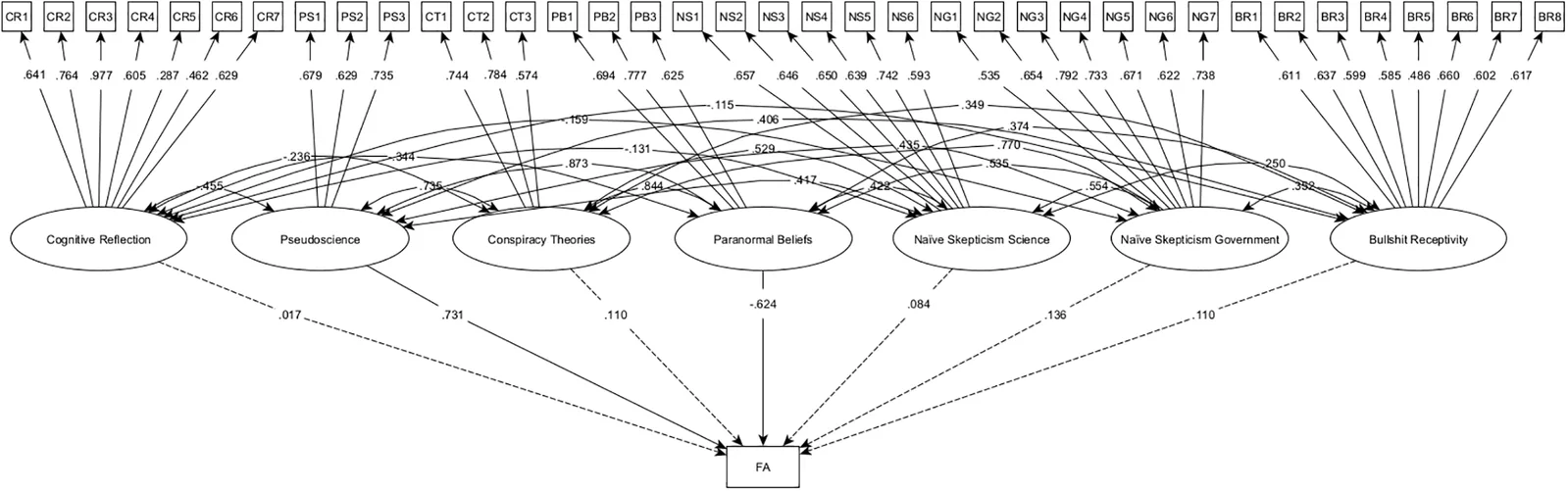
Covariate model: False-alarm rate. FA = False-alarm rate.

**Fig 3:**
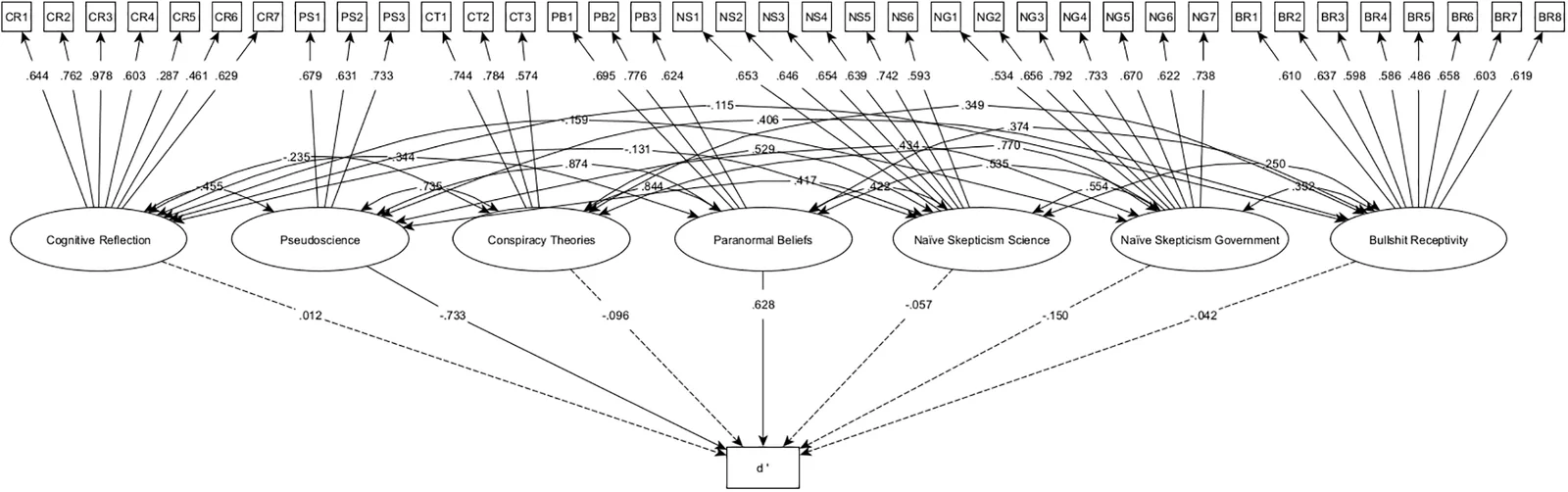
Covariate model: Discrimination sensitivity. $d^{\prime }$ = Discrimination Sensitivity.

Finally, to establish an explicative model of the joint effects, we estimated two structural equation models with correlated latent variables: one with the false alarm-rate as the dependent variable (Fig 2) and another with discrimination sensitivity as the dependent variable (Fig 3). In the case of the first model, the individual differences dimensions explain jointly a 25.8% of the variability in the false alarm-rate, showing adequate levels of fit ($\chi^{2}(285)=1331.296$; CFI = 0.941; TLI = 0.934; RMSEA = 0.038 [90%CI, 0.035–0.041]; and SRMR = 0.056). In the case of the second model, the individual differences dimensions explain jointly a 22.1% of the variability in the false alarm-rate, showing adequate levels of fit ($\chi^{2}(285)=1255.102$; CFI = 0.940; TLI = 0.934; RMSEA = 0.039 [90%CI, 0.036–0.042]; and SRMR = 0.057). We note that in both covariate models, we found suppression effects, in particular, negative suppression. This type of suppression occurs when the independent variables are highly correlated, which can cancel or reverse their effects on the dependent variable [55,56]. This prevented us from interpreting the specific effects of the predictors of both covariate models. Instead, we refer the reader to Table 3 for the univariate effects (see previous three paragraphs).

## Discussion

The current study had two goals. In the first place, we aimed to measure the influence of working memory load on people’s susceptibility to health-related misinformation. More specifically, we manipulated working memory load through a digit recall task to elicit conditions in which participants were prone to either Type 1 or Type 2 reasoning when judging the veracity of COVID-19 headlines. We found that the false-alarm rate in the working-memory load condition was comparable to that in the no-working-memory load condition. Importantly, the number of digits recalled was lower in the loaded condition, and response times to the COVID-19 headlines were higher, suggesting that the working memory manipulation affected task demands. However, this did not translate into a detectable effect on false-alarm rates in the COVID-19 headlines task. Nonetheless, this pattern should be interpreted with caution, as differences in response time do not necessarily provide direct evidence of shifts in Type 1 versus Type 2 processing.

In the second place, we investigated whether the individual-difference dimensions cognitive reflection, epistemically unwarranted beliefs (pseudoscience, conspiracy theories, paranormal beliefs), naïve skepticism (with regards to science and government), and bullshit receptivity were associated with health-related misinformation susceptibility. In concrete terms, using an SDT approach, we related these variables to the false alarm rate, discrimination sensitivity, and response bias. We found that cognitive reflection had a small negative relation with people’s false-alarm rates. Pseudoscience, conspiracy theories, paranormal beliefs, naïve skepticism-science, naïve skepticism-government, and bullshit receptivity showed all positive associations with people’s false-alarm rates. Our SEM analysis suggests that these associations are primarily driven by the individual-difference dimensions’ associations with discrimination sensitivity. Furthermore, we found only small associations between pseudoscience, conspiracy theories, naïve skepticism-science, and bullshit receptivity with response bias. Although these effects were generally modest in magnitude, they are consistent with prior work and contribute to refining existing accounts by highlighting the relative importance of discrimination sensitivity, rather than response bias, in explaining individual differences in misinformation susceptibility.

Regarding our first goal, our results are inconsistent with previous research using time pressure to manipulate cognitive elaboration [11,12,21,40]. We note that, following previous research on the effects of working memory load on reasoning and decision making [41,42], our working memory load manipulation was targeted to, in fact, load working memory instead of not giving participants enough time to engage in rigorous reasoning. Furthermore, as previously mentioned, we found evidence of the effectiveness of the working-memory manipulation (i.e., response times and digit recall). Yet it is possible that the current manipulation was insufficient to interfere with the deeper evaluative processes underlying credibility judgments, and it should be noted that the use of working memory load as a proxy for Type 1 versus Type 2 processing was not directly validated in the present study.

Importantly, discrimination performance was high ($d^{\prime }\approx 2.8$), and false-alarm rates were low (M≈.09), suggesting that participants were generally able to distinguish between true and false headlines with relative ease. Under such conditions, the task may have placed limited demands on effortful Type 2 processing, thereby reducing the likelihood that additional cognitive load would meaningfully influence performance. This raises the possibility of ceiling effects and restricted variance in the outcome measures, which may have attenuated observable effects and reduced statistical sensitivity. Accordingly, the absence of a detectable effect of cognitive load should not be taken as evidence that such effects are absent, as it may instead reflect characteristics of the task and measurement. Future research should investigate the effects of alternative cognitive load paradigms on misinformation susceptibility, particularly in contexts where stimuli are more ambiguous or calibrated to vary in difficulty.

Regarding our second goal, our SDT approach allowed us to better characterize the complex relationships between our individual-difference dimensions and misinformation susceptibility. For example, SDT predicts that high levels of naïve skepticism should produce more false alarms for belief-congruent information and more misses for belief-incongruent information, which should result in a negative relationship with discrimination sensitivity. An analysis based solely on overall accuracy rates would not have captured this relationship (for example, see [39]). Instead, by relating our individual-difference dimensions to false alarm rates, discrimination sensitivity, and response bias, we established that these dimensions exerted their influence on people’s tendency to judge misinformation as true primarily through discrimination sensitivity. In our sample, the most important factor influencing discrimination sensitivity was pseudoscience, which highlights the close relationship between these beliefs and susceptibility to health-related misinformation.

In our sample, the most important factor influencing discrimination sensitivity was pseudoscience, which highlights the close relationship between these beliefs and susceptibility to health-related misinformation. However, the observed effects were modest in magnitude and broadly consistent with prior findings. As such, the present results are best understood as refining existing accounts, with the analysis based on the SDT framework providing additional insight by distinguishing between discrimination sensitivity and response bias as underlying components of misinformation susceptibility.

Consistent with previous research using an SDT framework, we only found small associations between our individual-difference dimensions and response bias [13]. This is despite the fact that our analysis replicated previous research showing that both discrimination sensitivity and response bias predict people’s tendency to judge misinformation as true [11–13]. In this regard, it is important to note that the influence of response bias is conditional on the congruence of the information being processed with prior beliefs. In particular, Nahon et al [12] showed that anti-COVID-19-vaccine belief bias (defined as the difference between response bias for pro- and anti-COVID-19-vaccine statements) was a stronger predictor of susceptibility to misinformation than discrimination sensitivity. Critically, calculating anti-COVID-19-vaccine belief bias requires measuring participants’ attitudes towards COVID-19 vaccines beforehand (to classify them as pro- or anti-COVID-19-vaccine) and constructing pro- and anti-COVID-19-vaccine statements, which the current study did not do. Nonetheless, establishing robust relationships between individual-difference dimensions and response biases remains a challenge in this area of research. To the best of our knowledge, the only robust predictor of anti-COVID-19-vaccine belief bias is confidence in one’s beliefs [12]. Relatedly, the total number of COVID-19 headlines may have limited the variance of the SDT measures. Future research should aim to provide more headlines that include pro- or anti-COVID-19-vaccine statements. Additionally, the headlines should be pretested for plausibility, emotional valence, and prior exposure.

An additional limitation of our study was that our sample was homogeneous, consisting primarily of university students, for whom lower susceptibility to misinformation could be expected [57]. Therefore, our sample may have exhibited higher cognitive ability and lower susceptibility to misinformation than the general population, as reflected in the low false-alarm rates observed. This may have reduced the variability in susceptibility to health-related misinformation and/or cognitive reflection, which could explain the small associations found between cognitive reflection and false-alarm rates and between cognitive reflection and discrimination sensitivity. Restricted variance may also have limited the ability to detect the potential effect of cognitive load. Future research should aim to include more diverse populations across educational, age, and sociocultural backgrounds to better understand the broader applicability of these individual-difference predictors. Relatedly, performing subgroup comparisons of the pattern of results reported in this work is another important direction for future research.

A methodological consideration concerns the use of the parametric SDT measures $d^{\prime }$ and *c*, which assume that the underlying evidence distributions for true and false headlines are approximately normal and have equal variance. Because participants provided binary true/false judgments rather than confidence ratings, it was not possible to construct receiver operating characteristic (ROC) curves and directly evaluate these assumptions. Nevertheless, our use of $d^{\prime }$ follows a growing body of misinformation research that has applied the same SDT framework to headline-veracity judgments using binary responses [10–13]. Importantly, the primary pattern of results observed in the current study, namely, that individual differences were more strongly related to discrimination sensitivity than to response bias, is consistent with these previous studies. Future research would benefit from collecting confidence judgments and estimating ROC-based measures, as well as reporting complementary indices of sensitivity [58], to examine the robustness of conclusions across alternative SDT metrics.

## Conclusion

By applying an SDT framework, this study provides valuable insights into the psychological mechanisms underlying susceptibility to health-related misinformation. Our findings suggest that individual-difference dimensions —particularly beliefs in pseudoscience, conspiracy theories, paranormal phenomena, as well as levels of cognitive reflection, naïve skepticism, and bullshit receptivity— are associated with people’s ability to distinguish between true and false health information. These associations were especially robust with respect to discrimination sensitivity, suggesting that a person’s ability to discern the veracity of health information may be influenced by stable cognitive and epistemic dispositions.

## Supporting information

S1 File COVID-19 headlines. Full list of headlines (translated from Spanish).
(PDF)

S2 File CRT measurement-model results. Cognitive reflection test measurement-model results, including factor loadings.
(PDF)
