# Supplementary material for: Cognitive load and individual differences as drivers of health-related misinformation: A signal detection approach
Source: PLoS One. 2026 Aug 28;21(8):e0356642. doi: 10.1371/journal.pone.0356642 (PMC13524281; doi:10.1371/journal.pone.0356642)
Supplement: Supplementary file 1 [file pone.0356642.s001.pdf]

Supplementary material: COVID-19 Headlines

Note: The original items were written in Spanish.

1. The PCR test does not detect the presence of COVID-19 (0).
2. COVID-19 vaccine may cause infertility (0).
3. Asymptomatic people do not spread COVID-19 (0).
4. CoronaVac is produced from cells of aborted babies (0).
5. COVID-19 vaccine creates magnetic attraction (0).
6. The patent for testing COVID-19 was registered in 2015 (0).
7. New study shows that PCR test can cause Alzheimer's (0).
8. The light from body thermometers can hurt your eyes (0).
9. Authorized COVID-19 vaccines contain graphene (0).
10. Coronavirus is transmitted by 5G electromagnetic waves (0).
11. Those vaccinated against COVID-19 cannot donate blood or organs (0).
12. The plan for the pandemic has been designed since 2017 at the World Bank. (0).
13. The COVID-19 vaccine modifies the molecular structure of your DNA (0).
14. Chlorine dioxide is used to treat patients who test positive for COVID-19 (0).
15. PCR test swabs are sterilized with a powerful carcinogen (0).
16. Scientific studies show that masks are harmful and useless (0).
17. PCR testing is effective in detecting COVID-19 (1).
18. COVID-19 is caused by a virus, not a bacteria (1).
19. Masks are effective in preventing the spread of COVID-19 (1).
20. The use of masks has been key in preventing infection (1).
21. COVID-19 is a disease that can affect different organs (1).
22. Wearing a mask also reduces the risk of contracting influenza (1).
23. Age is one of the main risk factors for COVID-19 (1).

24. SARS-CoV-2 causes severe acute respiratory infection in some cases (1).
25. Isolation is a measure that allows us to reduce the number of infections (1).
26. Those who become infected with COVID-19 generate an immune response to the virus (1).
27. Vaccines have been highly effective in reducing infection rates (1).
28. The primary route of transmission of COVID-19 is through airborne droplets (1).
29. COVID-19 symptoms may persist for months after initial infection (1).
30. COVID-19 spreads most easily through direct contact between people (1).
31. Common symptoms of COVID-19 include fever, cough, fatigue and shortness of breath (1).
32. Wearing a mask reduces the chances of contracting COVID-19 by 66% (1).
