# Supplementary material for: Cognitive load and individual differences as drivers of health-related misinformation: A signal detection approach
Source: PLoS One. 2026 Aug 28;21(8):e0356642. doi: 10.1371/journal.pone.0356642 (PMC13524281; doi:10.1371/journal.pone.0356642)
Supplement: Supplementary file 2 [file pone.0356642.s002.pdf]

# Cognitive reflection test: Measurement-model results

A confirmatory factor analysis (CFA) was conducted to evaluate the unidimensional structure of the seven-item CRT measure. The model specified a single latent factor underlying all items. Model fit was excellent, $\chi^2(14) = 16.59$, $p = .279$, RMSEA = .017, 90% CI [.000, .044], CFI = .996, TLI = .995, and SRMR = .050, indicating that the hypothesized one-factor model provided an adequate representation of the data.

Standardized factor loadings are presented in Table 1. All loadings were statistically significant ($p < .001$). Most items showed moderate to strong loadings on the latent factor, ranging from .57 to .93. Item CRTCO5 exhibited a comparatively weak loading ($\lambda = .23$), suggesting that it was less strongly associated with the underlying construct than the remaining items.

Table 1: Standardized factor loadings for the one-factor CFA model

| Item | Standardized Loading ($\lambda$) |
|---|---|
| CRTCO1 | 0.687 |
| CRTCO2 | 0.724 |
| CRTCO3 | 0.926 |
| CRTCO4 | 0.651 |
| CRTCO5 | 0.229 |
| CRTCO6 | 0.574 |
| CRTCO7 | 0.671 |

Overall, the results support a unidimensional representation of the CRT measure, with the latent factor accounting for substantial variance in most items.
